# A case of refractory alopecia areata successfully treated by combining delgocitinib ointment with excimer laser

**DOI:** 10.1093/skinhd/vzaf013

**Published:** 2025-04-07

**Authors:** Yukito Kakeji, Toshiaki Kogame, Yosuke Yagi, Naotomo Kambe, Kenji Kabashima

**Affiliations:** Department of Dermatology, Osaka Red Cross Hospital, Osaka, Japan; Department of Dermatology, Kyoto University Graduate School of Medicine, Kyoto, Japan; Department of Dermatology, Osaka Red Cross Hospital, Osaka, Japan; Department of Dermatology, Kyoto University Graduate School of Medicine, Kyoto, Japan; Department of Dermatology, Kyoto University Graduate School of Medicine, Kyoto, Japan

## Abstract

Alopecia areata (AA) is an autoimmune disease that causes recurrent hair loss. No treatment has been effective in the long term because of the unstable efficacy and possible side effects. AA is primarily driven by Th1-type inflammation, centred around CD8^+^ T cells and interferon-γ (IFN-γ). Recent studies have revealed that the Janus kinase (JAK) family is also involved in the pathogenesis of AA, leading to JAK inhibitors emerging as a treatment for AA. We present a case of a 39-year-old Japanese woman with severe AA who exhibited a Severity of Alopecia Tool (SALT) score 80 accompanied by atopic dermatitis (AD). Despite conventional treatments, the condition worsened from a SALT score of 80 to 100. We subsequently attempted treatment with an excimer laser (EL), but no hair regrowth was observed. However, the introduction of 0.5% delgocitinib ointment in combination with EL led to complete hair regrowth beginning 2 months later, with complete remission achieved after 1 year. This case highlights the potential efficacy of combining delgocitinib ointment with EL in treating severe AA, particularly in patients with AD. The findings suggest that this combination therapy may provide a safer and more effective alternative to oral JAK inhibitors. Nevertheless, further studies are needed to elucidate the underlying mechanisms and fully evaluate the therapeutic synergy.

## Teaser

We report a case of a patient with refractory severe alopecia areata with comorbid atopic dermatitis who achieved complete remission through combination therapy with delgocitinib ointment and excimer laser. We discuss the combination of topical JAK inhibitors and excimer laser and believe the therapy may become an effective treatment option for patients with severe alopecia areata.

Alopecia areata (AA) is an autoimmune disease that causes recurrent hair loss and affects approximately 2% of the general population. AA treatment includes topical and systemic steroid therapy, excimer laser (EL) phototherapy using 308 nm ultraviolet B (UVB), and contact immunotherapy. AA is primarily driven by T helper (Th1)-type inflammation involving CD8^+^ T cells and interferon (IFN)-γ.^1^ AA can be associated with atopic dermatitis (AD) and often manifests severe alopecic symptoms. Furthermore, the involvement of the Janus kinase (JAK) family, consisting of JAK1, JAK2, JAK3 and Tyk2, in the development of AA has been reported.^1^ Therefore, oral JAK inhibitors such as baricitinib, a selective JAK1/JAK2 inhibitor, and ritlecitinib, a selective dual JAK3/TEC family kinase inhibitor, have emerged as available treatments for AA. As JAK inhibitors also suppress Th2 cytokines, including interleukin (IL)-4 and IL-13, they may also be effective for AD. Delgocitinib, a pan-JAK inhibitor, has been approved in Japan as an ointment for AD. Recently, dupilumab, a Th2 cytokine inhibitor, has been shown to achieve a Severity of Alopecia Tool (SALT) score of 30 or less by week 24 in 17.5% of patients with AA accompanied by AD.^2^ Although the relationship between AA and AD remains unclear, these findings suggest that topical formulations of JAK inhibitors could treat AA accompanied by AD. Herein, we report that the application of delgocitinib ointment with EL achieved complete remission of AA in a patient with AD.

## Case presentation

**Figure 1:**
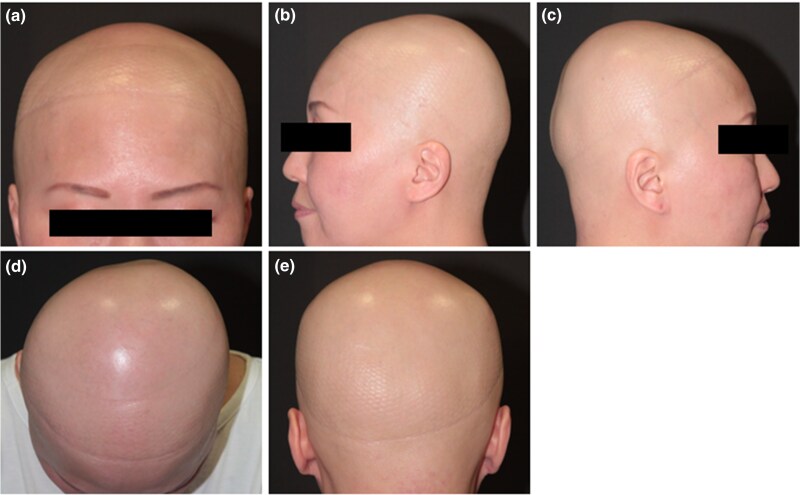
Clinical images: (a–e) frontal, left temporal, right temporal, parietal and occipital views of the scalp before combination therapy of delgocitinib ointment and excimer laser following the various treatments, including pulse corticosteroid therapy, contact immunotherapy and intralesional corticosteroid injections, were performed.

**Figure 2:**
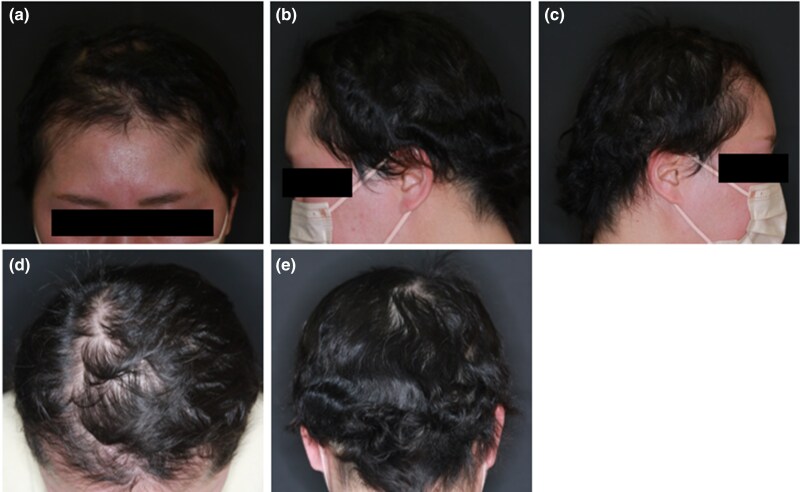
Clinical images after combination therapy: (a–e) frontal, left temporal, right temporal, parietal and occipital views of the scalp after combination therapy with delgocitinib ointment and excimer laser.

A 39-year-old Japanese woman with AD experienced hair loss 2 months ago. She had had AD since childhood, which was controlled by the strongest topical steroids and oral antihistamines. Despite prior treatment with the strongest topical steroid treatment, carpronium chloride and oral betamethasone once a day, her AA worsened, prompting a referral to our department. Physical examination was used to determine the SALT score. The body surface area involvement of AD was 0%. Laboratory tests revealed an elevated serum immunoglobulin E level (1403 IU/mL; normal <170 IU/mL). Dermoscopic examination revealed yellow and black dots, along with clustered short vellus hairs. Pathological examination of biopsy specimens exhibited decreased follicular density and a significant increase in telogen follicles number. Infiltration by inflammatory cells, predominantly lymphocytes, was observed around the follicular bulb. Pulse corticosteroid therapy was initiated, but no hair regrowth was observed (Table S1; see Supporting Information). Contact immunotherapy was discontinued after 2 months due to the development of contact dermatitis. Furthermore, intralesional injections of corticosteroids were administered for 1 year, but the SALT score worsened from 80 to 100 (Figure 1). Subsequently, EL was administered at a dose of 500 mJ/cm^2^, once a week for 6 months, without yielding hair regrowth. AA did not improve, even with several treatments. Additionally, erythema appeared on the vertex and posterior neck as AD symptoms during EL. Erythema was considered as aggravation of AD as it appeared 6 months after initiating EL. Therefore, 0.5% delgocitinib ointment was commenced in addition to EL. Remarkably, hair regrowth was observed after 2 months. One year after initiating the combination therapy, SALT score 0 was achieved, and no recurrence of AA was observed for 2 years (Figure 2). She is currently continuing treatment with delgocitinib monotherapy.

## Discussion

Severe alopecia is usually resistant to conventional treatments. This case report highlights refractory AA in a patient with AD successfully treated with a combination therapy of EL and delgocitinib ointment.

The oral JAK inhibitors baricitinib and ritlecitinib are currently available for the treatment of AA in Japan. Baricitinib, an oral selective JAK1/JAK2 inhibitor, and ritlecitinib, an oral selective dual JAK3/TEC family kinase inhibitor, showed that 31.3% and 14–31% of patients with severe AA with more than 50% scalp hair loss achieved a SALT score ≤ 20 at 24 weeks, respectively.^3,4^ Therefore, in theory, topical JAK inhibitors are expected to provide therapeutic effects for AA. Nonetheless, the 1.5% ruxolitinib cream did not show a visible effect for patients with AA, and delgocitinib ointment monotherapy was also demonstrated to be ineffective in moderate to severe AA.^5,6^ Although reports regarding the efficacy of topical JAK inhibitors for AA are still limited, tofacitinib was reported to show greater efficacy than ruxolitinib in both topical and oral formulations.^7^ Delgocitinib and tofacitinib are pan-JAK inhibitors, whereas ruxolitinib is a selective JAK1/JAK2 inhibitor. In addition, the effectiveness of ritlecitinib suggests an important role of JAK3 in the pathogenesis of AA. Therefore, delgocitinib and tofacitinib, which also inhibit JAK3, may theoretically exhibit higher efficacy in AA than ruxolitinib.

EL exerts its therapeutic effects on AA by its immunomodulatory properties via CD8^+^ T-cell apoptosis and regulatory T-cell (Treg) infiltration.^8^ Furthermore, EL exerts its therapeutic effects on AA by extending the anagen phase through Wnt signalling in hair follicles.^9^ The efficacy of EL for AA has been investigated in several studies, and meta-analyses have reported its usefulness for patchy AA. However, EL was ineffective in all patients with severe AA in these studies.^10^ A patient with severe AA and vitiligo was reported to achieve partial response for AA through combination therapy with topical delgocitinib and EL. The SALT score improved from 100 to 30 over 15 months.^11^ Therefore, delgocitinib ointment may be effective for AA, particularly when combined with EL. Several reports described the recommended application of a combination of topical JAK inhibitors and EL therapy for treating vitiligo due to its effectiveness.^12^ However, molecular insights of combination therapy of JAK and EL are yet to be elucidated, especially for AA. Recent studies showed that Tregs are crucial for inducing hair regrowth from hair follicle stem cells and that Th2 cytokines suppress the regulatory functions of Tregs.^13,14^ In addition, the Th2 milieu is demonstrated to disturb the differentiation of hair follicle stem cells.^15^ As mentioned above, EL activates Tregs. Thus, the treatment of AD with delgocitinib, which modifies the Th2 milieu, supports the activation of the regulatory function of Tregs, leading to the recovery of hair follicle stem cells.

Oral JAK inhibitors have shown significantly higher efficacy for AA than topical formulations.^7^ However, while oral JAK inhibitors raise concerns about risks of viral reactivation, malignancy and deep vein thrombosis,^1^ topical JAK inhibitors primarily cause localized side effects such as acne. Therefore, topical JAK inhibitors are considered safer in terms of side effects. Regarding the combination therapy of topical JAK inhibitors and EL, the immunosuppressive effects of JAK inhibitors could potentially increase the risk of developing skin tumours due to UV exposure from EL treatment. Although serious side effects of the combination therapy have not been reported, careful monitoring of the patient’s skin is required.

Our present case suggests that combination therapy with delgocitinib ointment and EL can be effective even for severe AA and could be a theoretically safer alternative to oral JAK inhibitors.

## Supplementary Material

vzaf013_Supplementary_Data

## Data Availability

No new data were generated or analysed in support of this research.
