# Supplementary material for: A case of refractory alopecia areata successfully treated by combining delgocitinib ointment with excimer laser
Source: Skin Health Dis. 2025 Apr 7;5(2):154–7. doi: 10.1093/skinhd/vzaf013 (PMC12068471; doi:10.1093/skinhd/vzaf013)
Supplement: vzaf013_Supplementary_Data [file vzaf013_supplementary_data.docx]

**TABLE S1**


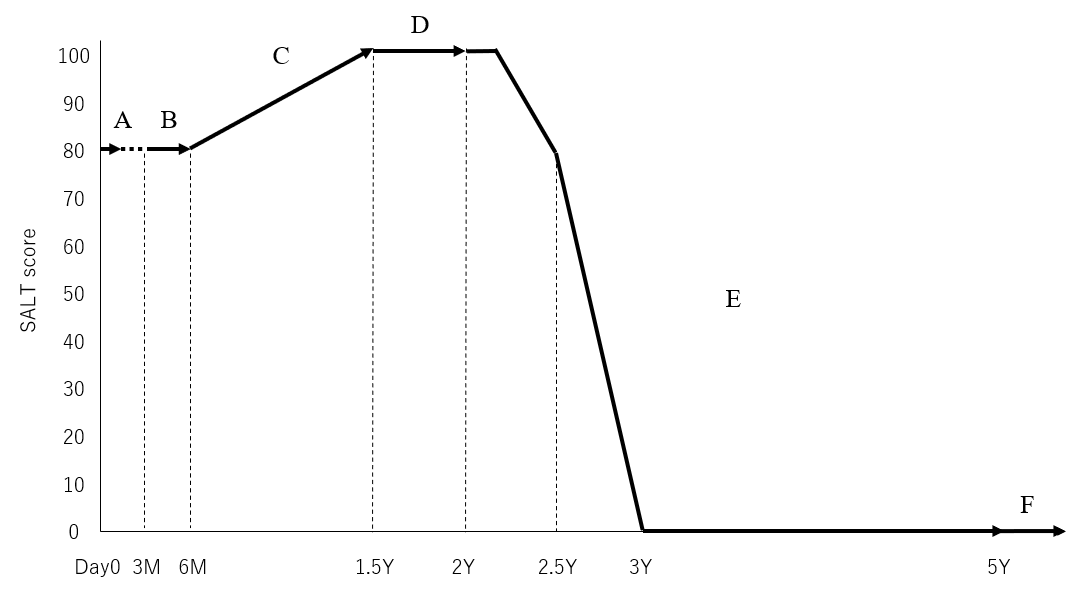


Table S 1. Change of treatment and SALT score:

**A:** **pulse corticosteroid therapy** -methylprednisolone at 500 mg/day for 3 days.

**B**: **contact immunotherapy** - diphenylcyclopropenone, administered once every 2 weeks for 2 months.

**C**: **intralesional corticosteroid injections -** 0.2% corticosteroid, administered monthly for 1 year.

**D**: **excimer laser phototherapy** - 500 mJ/cm^2^, once a week for 6 months.

**E**: **combination therapy** – excimer laser phototherapy **(** 500 mJ/cm^2^ once a week ) and 0.5% delgocitinib ointment ( applied twice daily ) for 3 years.

**F**: **0.5% delgocitinib ointment** – applied twice daily.
